# InGaP (GaInP) mesa p-i-n photodiodes for X-ray photon counting spectroscopy

**DOI:** 10.1038/s41598-017-10502-y

**Published:** 2017-08-31

**Authors:** S. Butera, G. Lioliou, A. B. Krysa, A. M. Barnett

**Affiliations:** 1 Semiconductor Materials and Devices Laboratory, School of Engineering and Informatics, University of Sussex, Brighton, BN1 9QT UK; 2 EPSRC National Centre for III-V Technologies, University of Sheffield, Mappin Street, Sheffield, S1 3JD UK

## Abstract

In this paper, for the first time an InGaP (GaInP) photon counting X-ray photodiode has been developed and shown to be suitable for photon counting X-ray spectroscopy when coupled to a low-noise charge-sensitive preamplifier. The characterisation of two randomly selected 200 μm diameter and two randomly selected 400 μm diameter In_0.5_Ga_0.5_P p^+^-i-n^+^ mesa photodiodes is reported; the i-layer of the p^+^-i-n^+^ structure was 5 μm thick. At room temperature, and under illumination from an ^55^Fe radioisotope X-ray source, X-ray spectra were accumulated; the best spectrometer energy resolution (FWHM) achieved at 5.9 keV was 900 eV for the 200 μm In_0.5_Ga_0.5_P diameter devices at reverse biases above 5 V. System noise analysis was also carried out and the different noise contributions were computed.

## Introduction

In_0.5_Ga_0.5_P (Ga_0.5_In_0.5_P) is a direct wide bandgap (~1.9 eV at room temperature^1–3^) ternary compound with a density of 4.5 gcm^−3^ and high X-ray and γ-ray linear absorption coefficients^4, 5^. Its crystalline lattice parameter nearly matches that of GaAs which is commonly used as a substrate material in epitaxy. This allows high quality epitaxial growth of relatively thick InGaP-based structures used in optoelectronics, mainly, in visible light emitting devices and solar cells. The combinations of the above properties make In_0.5_Ga_0.5_P also potentially attractive for applications as a detector material for X-ray and possibly γ-ray photon counting spectroscopy. Due to their low leakage currents, wide bandgap semiconductor X-ray spectrometers can operate at room temperature and above without cooling systems^6–8^. Such high temperature (≥20 °C) operation may provide benefits due to the reduced mass, volume, and power requirements of such technologies brought by the elimination of cooling systems. Consequently, wide bandgap materials are attractive choices for the development of low-cost, compact and temperature tolerant X-ray spectrometers that may be useful in space missions^9–11^, and for terrestrial applications outside the laboratory environment, such as industrial monitoring, defence and security, and underwater exploration^12, 13^. Other wide bandgap detector technologies for X-ray spectrometers include SiC^6^, GaAs^7, 14^, Al_0.52_In_0.48_P^8, 15, 16^, AlGaAs^17^, CdTe^18–20^, and CdZnTe^18, 21–23^.

In_0.5_Ga_0.5_P combines the properties of its binary relations, GaP and InP. Moreover, (advantageously compared with AlInP) it does not include Aluminium, which, along with silicon, is a material frequently of interest in planetary X-ray fluorescence spectroscopy (XRF). Detectors without these materials are thus desirable in order to reduce the complexity of spectral analysis through the removal of these lines from the detector’s self-fluorescence. Because of the higher X-ray linear attenuation coefficients of In_0.5_Ga_0.5_P compared to those of some other wide bandgap materials (e.g. GaAs, SiC, AlGaAs, and AlInP), comparatively thinner In_0.5_Ga_0.5_P detectors can be produced to obtain the same quantum efficiency. Further, improved high temperature performance can be achieved, not only because of the wide bandgap but also because of the smaller volume of semiconductor material required in the detector.

The use of In_0.5_Ga_0.5_P for X-ray spectroscopy is a new research field that may provide innovative X-ray detection instrumentation with excellent characteristics. The results reported in this paper are the first detection of X-rays with InGaP and the first demonstration of its suitability for photon counting X-ray spectroscopy. These results are particularly significant since GaP and InP were found to be not spectroscopic at room temperature^24–27^. 200 μm and 400 diameter non-avalanche In_0.5_Ga_0.5_P photodiodes were connected to custom low-noise charge-sensitive preamplifier electronics developed at our laboratory in order to produce an X-ray spectrometer. A system energy resolution of 900 eV at 5.9 keV was found for a randomly selected 200 μm device at reverse biases above 5 V. The work is of potential importance for the development of wide bandgap X-ray and γ-ray spectrometers for planetary and space science missions to extreme environments (such as Mercury, Venus, Jupiter, and Saturn), for space science instrumentation to study the near Sun environment, as well as for use in harsh terrestrial environments.

## Results

Two 200 μm (devices D1 and D2) and two 400 μm diameter (devices D3 and D4) In_0.5_Ga_0.5_P photodiodes were studied in this work; the growth and the fabrication details are given in the Methods section. For the areas of the photodiodes not covered by the top contact, X-ray quantum efficiencies (*QE*) of 53% and 44% were calculated at energies of 5.9 keV and 6.49 keV, respectively, using the Beer-Lambert law and assuming complete charge collection in the p and i layers. For the areas covered by the top contact this reduced to 44% and 38%, respectively. Considering the portion of top contacts covering the top surfaces of the 400 μm and the 200 μm diameter photodiodes, total quantum efficiencies of 50% at 5.9 keV and 42% at 6.49 keV were obtained for the 400 μm device, and total quantum efficiencies of 49% at 5.9 keV and 41% 6.49 keV were found for the 200 μm device. The linear attenuation coefficients used in the *QE* calculations were 0.145 μm^−1^ and 0.112 μm^−1^ at 5.9 keV and 6.49 keV, respectively^4, 5, 28^; these values are higher than many other semiconductors such as Si^28^, SiC^4^, GaAs^4^, and Al_0.52_In_0.48_P^15^, but lower than for CdZnTe^5, 28^. The calculated total *QE* values are in accordance with those experimentally determined in the photocurrent measurements with an ^55^Fe radioisotope X-ray source.

### Electrical characterisation

**Figure 1:**
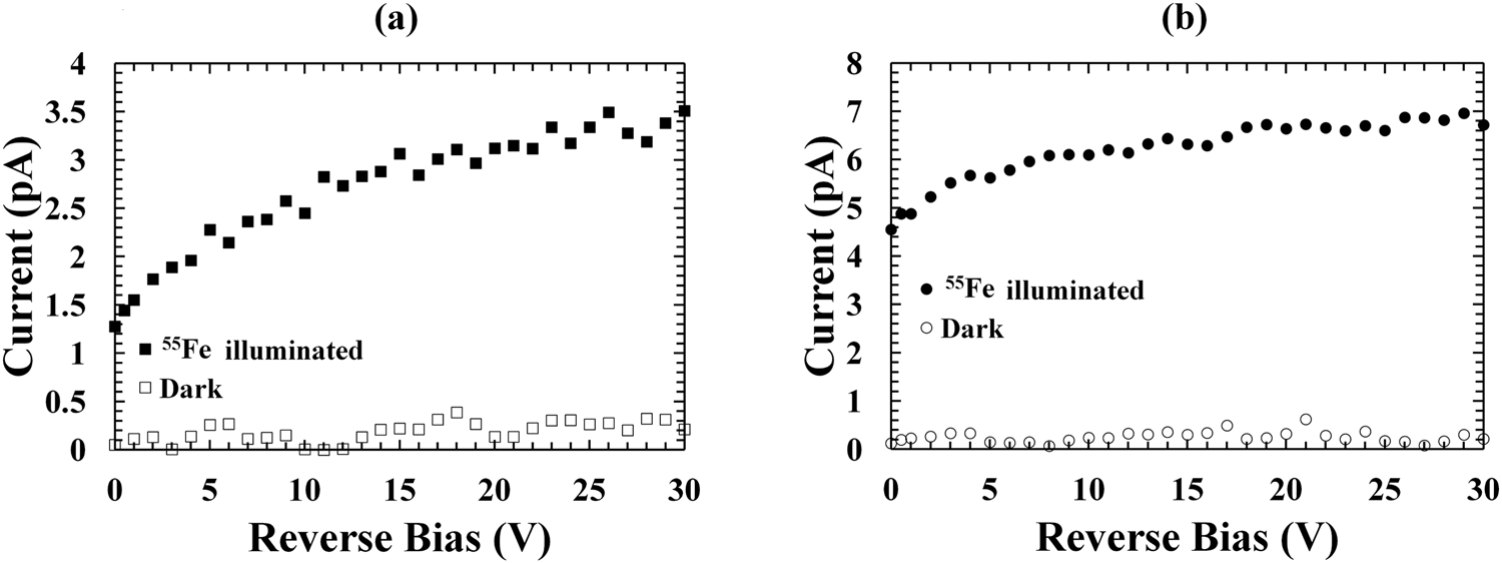
Dark (empty symbols) and ^55^Fe illuminated (filled symbols) current measurements as functions of applied reverse bias for the (**a**) 200 µm diameter, D1 (squares), and (**b**) 400 µm diameter, D3 (circles) In_0.5_Ga_0.5_P devices at room temperature.

The currents of the In_0.5_Ga_0.5_P devices were studied as functions of applied reverse bias from 0 V to 30 V in dark conditions, and under the illumination of an ^55^Fe radioisotope X-ray source (Mn Kα = 5.9 keV, Mn Kβ = 6.49 keV). The In_0.5_Ga_0.5_P photodiodes were investigated at room temperature in a dry nitrogen atmosphere (relative humidity <5%). A Keithley 6487 picoammeter/voltage source was used during the experiment; the uncertainty associated with the current readings was 0.3% of their values plus 400 fA, while the uncertainty associated with the applied biases was 0.1% of their values plus 1 mV^29^. Figure 1 shows the dark and the illuminated current curves as a function of reverse bias for 200 µm D1 (a) and 400 µm D3 (b). Similar results were found for D2 and D4. For all the photodiodes, dark current values < 0.22 pA were measured across the reverse bias range investigated (up to 30 V) (corresponding to current densities of 6.7 × 10^−10^ A/cm^2^ and 1.7 × 10^−10^ A/cm^2^ for the 200 μm and 400 μm diameter devices, respectively). The illuminated current measurements were taken when the ^55^Fe radioisotope X-ray source was positioned 6 mm above the top of each In_0.5_Ga_0.5_P mesa photodiode. Illuminated currents of 3.5 pA and 7 pA were observed at 30 V for the 200 μm and the 400 μm diameter In_0.5_Ga_0.5_P photodiodes, respectively. Subtracting the illuminated currents from the dark currents, photocurrents of 3.3 pA and 6.5 pA were calculated at 30 V for the 200 μm and the 400 μm diameter devices, respectively.

**Figure 2:**
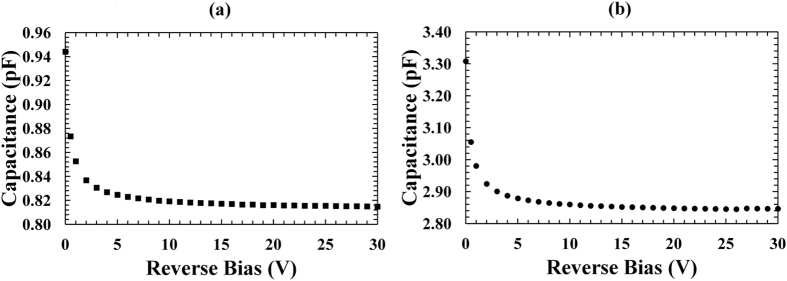
Capacitance measurements as a function of applied reverse bias for the In_0.5_Ga_0.5_P devices at room temperature. (**a**) 200 μm diameter device, D1 (filled squares); (**b**) 400 μm diameter device, D3 (filled circles).

Capacitance measurements of the In_0.5_Ga_0.5_P devices were made as a function of applied reverse bias from 0 V to 30 V using an HP 4275 A Multi Frequency LCR meter. The test signal was sinusoidal with a 50 mV rms magnitude and a 1 MHz frequency. The uncertainty associated with each capacitance reading was ~0.12% plus an experimental repeatability error of (±0.07 pF); the uncertainty associated with the applied biases was 0.1% of their values plus 1 mV^30^. The capacitance of an identical empty package was also measured and subtracted from the measured capacitance of each packaged photodiode to determine the capacitances of the devices themselves. Figure 2 shows the capacitance as a function of applied reverse bias for D1 (a) and D3 (b). The results for D2 and D4 were so similar as to be indistinguishable from those presented.

The depletion depth (*W*) of each diode was then calculated by:

1 $$W=\frac{{\varepsilon }_{0}{\varepsilon }_{r}A\,}{C},$$

where *ε*_0_ was the permittivity of the vacuum, *ε*_*r*_ was the In_0.5_Ga_0.5_P dielectric constant (11.7^31^), and *A* was the device area^32^.

**Figure 3:**
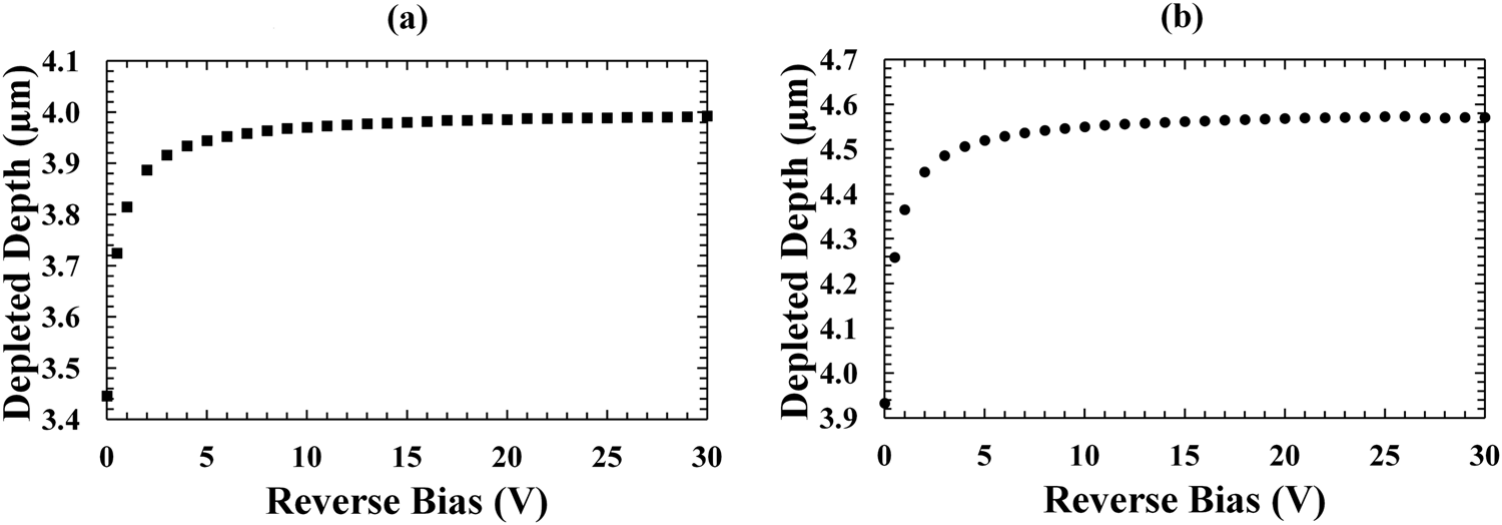
Depletion depth as a function of applied reverse bias for the In_0.5_Ga_0.5_P devices at room temperature. (**a**) 200 μm devices, D1 (filled squares); (**b**) 400 μm devices, D3 (filled circles).

Figure 3 shows the depletion depths as functions of applied reverse bias for D1 (a) and D3 (b), respectively. The results for D2 and D4 were so similar as to be indistinguishable from those presented.

At low reverse biases, the depletion depth increased as the reverse bias was increased; above 5 V the depletion depth remained almost constant in all the diodes analysed, this was due to the i-layer being fully swept-out at these biases. At 30 V, depletion depths of (4.0 ± 0.5) μm and (4.6 ± 0.2) μm were calculated from the capacitance measurements for the 200 μm and 400 μm diameter devices, respectively.

**Figure 4:**
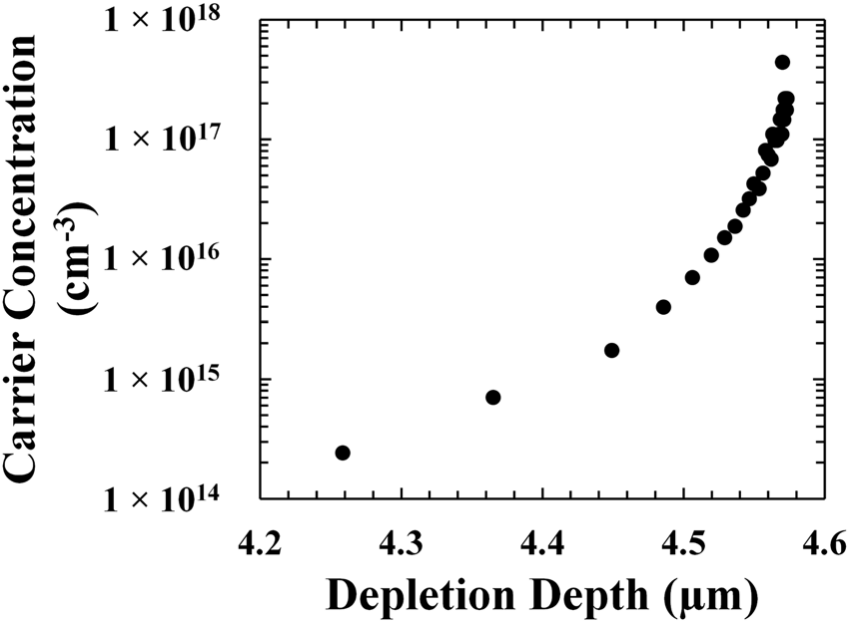
Doping concentration below the p^+^-i junction as a function of depletion depth at room temperature for a 400 μm diameter In_0.5_Ga_0.5_P device (D3).

The doping concentration (*N*) below the p^+^-i junction as a function of depletion depth (*W*) was calculated by:

2 $$N(W)=\frac{2}{q{\varepsilon }_{0}{\varepsilon }_{r}{A}^{2}}(\frac{dV}{d[1/{C}^{2}]}),$$

where *ε*_0_ was the permittivity of the vacuum, *ε*_*r*_ was the relative permittivity of In_0.5_Ga_0.5_P (11.7^31^), and *A* was the device area^32^. Figure 4 shows the obtained doping concentration for a representative 400 μm diameter In_0.5_Ga_0.5_P device, D3. The doping density in the i-layer was 2 × 10^14^ cm^−3^, such value increased to 4 × 10^17^ cm^−3^ at the i-n interface.

### X-ray spectroscopy and noise analysis

**Figure 5:**
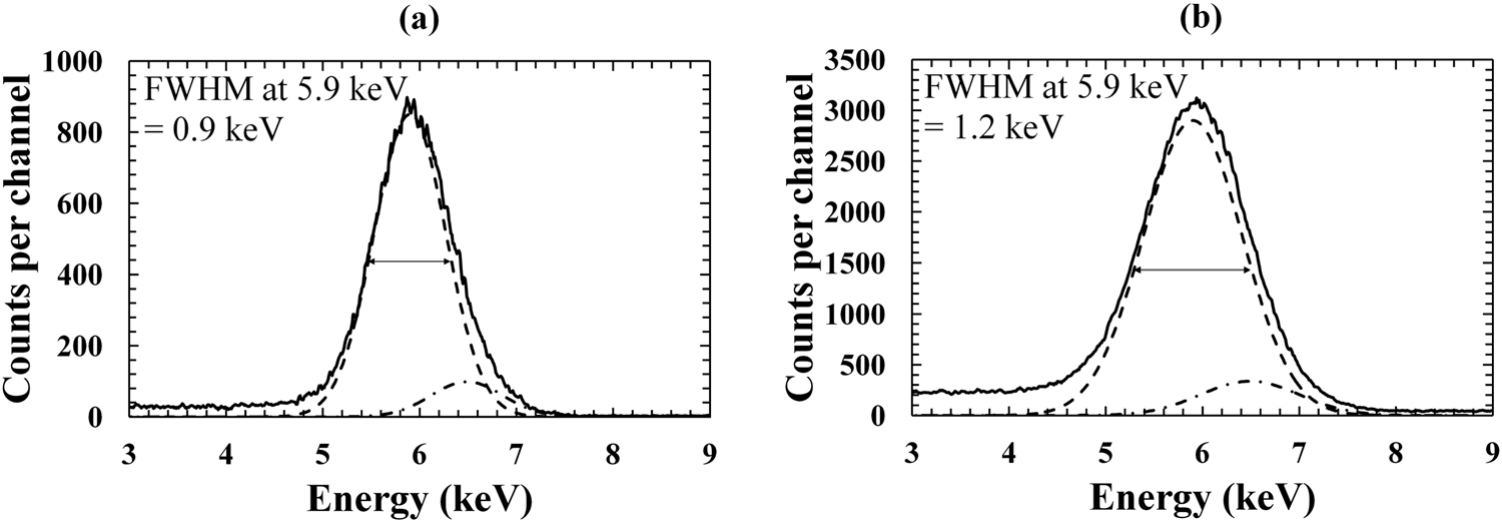
^55^Fe X-ray spectrum accumulated at 5 V reverse bias using the In_0.5_Ga_0.5_P devices: (**a**) 200 µm diameter device, D1; (**b**) 400 µm diameter device, D3.

X-ray spectra were collected using the 200 μm and 400 μm diameter devices and an ^55^Fe radioisotope X-ray source. As per the photocurrent measurements, the distance between the top surface of the In_0.5_Ga_0.5_P photodiodes and the X-ray source was 6 mm. A custom-made low-noise charge-sensitive preamplifier of feedback resistorless design, similar to ref. 33, was connected to each In_0.5_Ga_0.5_P diode in turn. The signal from the preamplifier was amplified and shaped using an Ortec 572a shaping amplifier, the output of which was connected to an Ortec Easy-MCA-8K multichannel analyser. Spectra were accumulated with the In_0.5_Ga_0.5_P diodes reverse biased at 0 V, 5 V, 10 V and 15 V; a shaping time of 10 μs and a live time limit of 100 s for each spectrum were used. Figure 5 shows the X-ray spectra obtained at 5 V with D1 (a) and D3 (b), respectively. Similar results were found for D2 and D4.

In each spectrum, the observed ^55^Fe photopeak was the combination of the Mn Kα and Mn Kβ lines at 5.9 keV and 6.49 keV, respectively. Gaussians were fitted to the combined peak, taking into account the relative X-ray emission rates of the ^55^Fe radioisotope X-ray source at 5.9 keV and 6.49 keV in the appropriate ratio^34^ and the relative difference in efficiency of the detector at these X-ray energies. The In_0.5_Ga_0.5_P spectrometer energy resolution, as quantified by the FWHM at 5.9 keV, was studied as a function of detector reverse bias. At 0 V, the FWHM at 5.9 keV was the poorest obtained (FWHM at 5.9 keV of 1 keV and 1.4 keV were observed for both the 200 μm (D1 and D2) and the 400 μm diameter (D3 and D4) devices, respectively), this was due to the increased contribution of incomplete charge collection noise which reduced at higher reverse biases. At 5 V and above, the charge collection efficiency was increased (the incomplete charge collection noise decreased) and the FWHM at 5.9 keV improved. The peak channel position and the FWHM remained constant at reverse biases ≥5 V, indicating that a charge collection efficiency (CCE) of 1 was obtained for each In_0.5_Ga_0.5_P device within the bias range investigated. At 5 V reverse bias, FWHM at 5.9 keV of 0.9 keV and 1.2 keV were observed for both the 200 μm (D1 and D2) and the 400 μm diameter (D3 and D4) devices, respectively.

**Figure 6:**
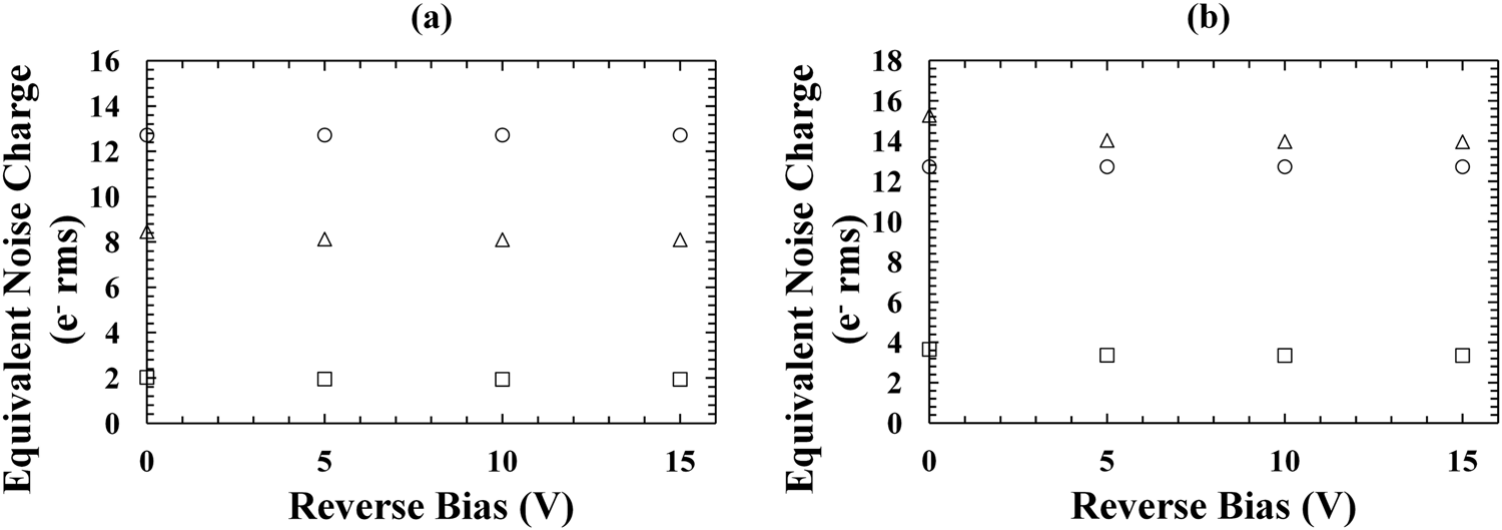
Equivalent noise charge as a function of reverse bias using the In_0.5_Ga_0.5_P devices: (**a**) 200 µm diameter device, D1; (**b**) 400 µm diameter device, D3. In both graphs, the parallel white noise (empty circles), the series white noise adjusted for induced gate current noise (empty triangles), and the 1/*f* noise (empty squares) contributions are shown.

Noise analyses were carried out in order to identify the different noise contributions that contributing to FWHM broadening. The spectral resolution of a non-avalanche photodiode X-ray spectrometer is given by:

3 $${\rm{\Delta }}E[eV]=2.355\omega \sqrt{\frac{FE}{\omega }+{R}^{2}+{A}^{2}},$$

where *ΔE* is the FWHM, *ω* is the electron-hole pair creation energy, *F* is the Fano factor, *E* is the energy of the X-ray photon absorbed, and *R* and *A* are the electronic noise and the incomplete charge collection noise, respectively^35^. The fundamental “Fano limited” energy resolution (i.e. *R* = 0 and *A* = 0) for In_0.5_Ga_0.5_P was estimated to be 137 eV at 5.﻿9 keV, assuming an In_0.5_Ga_0.5_P electron-hole pair creation energy of 4.8 eV (2.5 times the bandgap) and a Fano factor of 0.12. This noise contribution takes into account the statistical nature of the ionization process in a semiconductor X-ray detector. Since the measured FWHM was bigger than 137 eV, it was essential to consider the contributions from the other noise sources. The electronic noise of the system consists of parallel white noise, series white noise, induced gate current noise, 1/*f* noise, and dielectric noise^35–37^. The leakage currents of the detector and input JFET of the preamplifier are drivers of the parallel white noise; whilst the capacitances of the detector and input JFET of the preamplifier determine the series white noise and 1/*f* noise^35, 36^. Figure 6a and b show the calculated parallel white noise, series white noise, and 1/*f* noise as functions of detector reverse bias for a 200 μm (D1) and a 400 μm (D3) diameter devices, respectively. The series white noise was adjusted for induced gate current noise^35, 36^. In_0.5_Ga_0.5_P devices with same diameters had similar noise contributions.

**Figure 7:**
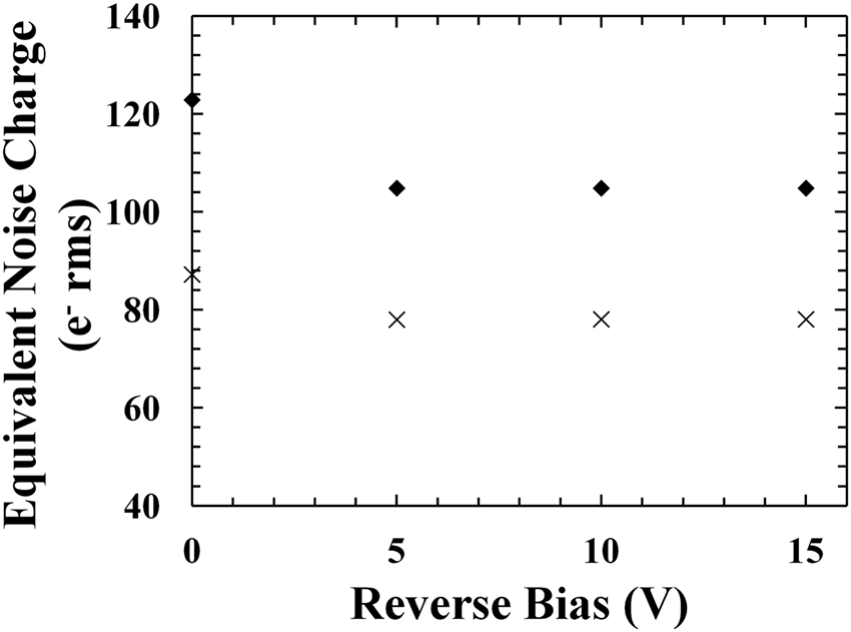
Equivalent noise charge of the dielectric noise and incomplete charge collection noise as a function of reverse bias using the In_0.5_Ga_0.5_P devices: 200 μm diameter device, D1 (crosses); 400 μm diameter device, D3 (filled rhombuses).

The parallel white noise contributions were similar for the 200 μm and the 400 μm diameter devices at each reverse bias analysed; this was due to similar dark currents in devices of both sizes, as shown in Fig. 1. In contrast, the series white noise and the 1/*f* noise were greater for the 400 μm diameter device compared with the 200 μm diameter device; this was due to the greater capacitance measured for the devices with bigger diameter, as shown in Fig. 2. The increased FWHM observed for the 400 μm diameter devices can be explained in part by considering the increased series white noise and the 1/*f* noise contributions. The Fano noise, the parallel white noise, the series white noise, and the 1/*f* noise contributions at 5.9 keV were then subtracted in quadrature from the measured FWHM at 5.9 keV in order to compute the combined contribution of the dielectric noise and incomplete charge collection noise at 5.9 keV. Figure 7 shows the combined equivalent noise charge of the dielectric noise and incomplete charge collection noise as a function of revers bias for the spectrometers with the In_0.5_Ga_0.5_P 200 μm device D1 and the In_0.5_Ga_0.5_P 400 μm device D3. Similar results were obtained for the spectrometers with D2 and D4.

The combined contribution of the dielectric noise and incomplete charge collection noise (added in quadrature) was greater using the 400 μm devices with respect to the 200 μm devices at all the reverse biases. At 0 V, the combined equivalent noise charge was 123 e^−^ rms and 87 e^−^ rms for the 400 μm devices and the 200 μm devices, respectively. At reverse biases ≥5 V, equivalent noise charge values of 105 e^−^ rms and 78 e^−^ rms were computed for the 400 μm devices and the 200 μm devices, respectively. Since the dielectric noise was independent of detector bias^35^, the difference in the values of the combined equivalent noise charge observed at 0 V compared with those at ≥5 V for each device can be attributed to incomplete charge collection noise at 0 V; thus it can be said that at 0 V there were 64 e^−^ rms and 39 e^−^ rms of incomplete charge collection noise using the 400 μm device and the 200 μm device, respectively, and that incomplete charge collection noise was insignificant at reverse biases ≥5 V.

In Fig. 7, the equivalent noise charge at reverse biases ≥5 V was only due to the dielectric contribution; the dielectric equivalent noise charge (*ENC*_*D*_) is given by:

4 $$EN{C}_{D}=\frac{1}{q}\sqrt{{A}_{2}2kTDC},$$

where q is the electric charge, *A*_2_ is a constant (1.18) depending on the type of signal shaping^37^, k is the Boltzmann constant, *D* is the dielectric dissipation factor, and *C* is the capacitance^35^. Using equation 4 and the experimental data reported in Fig. 7, an effective combined dielectric dissipation factor as high as (4.2 ± 0.4) × 10^−3^ was found for the lossy dielectrics; it should be noted that this does not correspond directly to the dissipation factor of In_0.5_Ga_0.5_P, rather it is indicative of the effective combined dissipation factor of all dielectrics contributing to this noise as it is analyzed here.

The dielectric noise shown in Fig. 7 takes into account a contribution due to the diode itself and a contribution due to the other dielectrics in the system. We assumed that the variation in dielectric noise observed between the spectrometer with the 400 μm diameter device (105 e^−^ rms) and the spectrometer with the 200 μm diameter device (78 e^−^ rms) was only due to the different diodes used; such variation was related, using equation 4, to the different diodes capacitances (2.85 pF for the 400 μm diameter device and 0.82 pF for the 200 μm diameter device, as shown in Fig. 2). The contribution of other dielectrics in the system was considered similar in both spectrometers. Under these assumptions, it was also possible to estimate the dielectric dissipation factor of In_0.5_Ga_0.5_P: a value of ∼6.5 × 10^−3^ was computed.

At room temperature, the energy resolution (FWHM) at 5.9 keV using the In_0.5_Ga_0.5_P devices were not as good as the best that have been reported for SiC (196 eV)^6^ and GaAs (266 eV)^14^. However, the very good performance reported in refs 6 and 14 was in a large part due to the lower electronic noise associated with the preamplifiers used (particularly due to the direct connection of the detectors to the preamplifer input transistors, compared with the use of a discrete wire-ended packaged transistor in the present work) as well very high quality semiconductor materials. FWHM similar to those reported here for In_0.5_Ga_0.5_P have been recently reported with an Al_0.52_In_0.48_P detector (FWHM at 5.9 keV of 0.93 keV for a 200 µm diameter Al_0.52_In_0.48_P device)^15^ where readout electronics similar to those used for the In_0.5_Ga_0.5_P were also used. The energy resolution achieved for with In_0.5_Ga_0.5_P photodiodes was better than those previously reported with Al_0.8_Ga_0.2_As detectors^17^, although the readout electronics used for the Al_0.8_Ga_0.2_As detectors were not of identical design as those used here. Very notably, the In_0.5_Ga_0.5_P detectors reported here perform significantly better than the corresponding binary compounds GaP and InP^24–27^: In_0.5_Ga_0.5_P was found to have high enough energy resolution to allow photon counting X-ray spectroscopy at room temperature, this is not true for GaP and InP. This paper is the first report of an In_0.5_Ga_0.5_P photon counting X-ray spectrometer; improved results, particularly in term of energy resolutions, are expected to be achieved in the future with further technology developments.

## Discussion

The results reported in this paper are the first demonstration of an In_0.5_Ga_0.5_P X-ray detector and the first demonstration of In_0.5_Ga_0.5_P used for a room temperature X-ray spectrometer. Although GaP and InP were previously reported to be not spectroscopic at room temperature^24^, In_0.5_Ga_0.5_P has been found to be suitable for photon counting X-ray spectroscopy. Under the illumination with ^55^Fe X-ray source and using custom-made low-noise charge sensitive preamplifier electronics developed at our laboratory, spectra were collected with 200 μm and 400 diameter non-avalanche In_0.5_Ga_0.5_P photodiodes reverse biased at 0 V, 5 V, 10 V and 15 V. A shaping time of 10 μs was used during the experiment. The best energy resolutions (FWHM) obtained at 5.9 keV were 0.9 keV and 1.2 keV using the 200 μm and 400 μm diameter devices, respectively, at 5 V. No change in FWHM was observed at reverse biases greater than 5 V, suggesting that incomplete charge collection noise was insignificant at these reverse biases. The greater FWHM observed with the 400 μm diameter devices compared with the 200 μm diameter devices can be explained considering the increased series white noise, 1/*f* noise, and dielectric noise contributions of the larger detector. Since the 400 μm diameter devices had greater capacitances than the 200 μm diameter devices, the series white and the 1/*f* noises were bigger in the 400 μm diameter devices. The parallel white noise, instead, was similar between all the diodes analysed due to similar (and very low, <0.4 pA) leakage currents. The contribution of the dielectric noise was greater for the spectrometer with the 400 μm devices (105 e^−^ rms) than the spectrometer with the 200 μm devices (78 e^−^ rms); this noise contribution was found to be the main source of noise limiting the spectrometers energy resolution. Assuming that the variation in dielectric noises observed between the spectrometer with the 400 μm diameter device and the spectrometer with the 200 μm diameter device was only due to the different diode capacitances, an In_0.5_Ga_0.5_P dissipation factor of ∼6.5 × 10^−3^ was also estimated.

## Method

### Device structure

**Figure 8:**
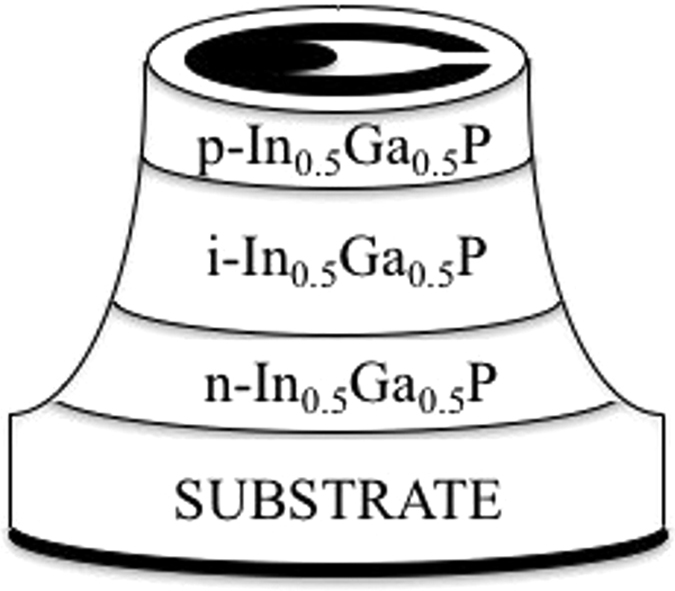
Diagram of an In_0.5_Ga_0.5_P mesa device; the In_0.5_Ga_0.5_P wafer was fully etched (i.e. down to the GaAs substrate) to form the mesa structure. The geometry of the top contact is also shown in the figure; the bottom contact covered the whole rear surface of the GaAs substrate.

The In_0.5_Ga_0.5_P structure was grown by metalorganic vapour phase epitaxy on a (100) n-GaAs substrate. The substrate’s epitaxial surface had a miscut angle of 10° towards 〈111〉 A, in order to suppress CuPt type ordering^38–40^. The latter phenomenon results in a reduction of the In_0.5_Ga_0.5_P bandgap, deterioration of the In_0.5_Ga_0.5_P crystalline quality and surface morphology, and, consequently, may deteriorate the spectral characteristics (energy resolution) of the fabricated devices. The InGaP n^+^ (0.1 μm), i (5 μm) and p^+^ (0.2 μm) layers were successively grown on the GaAs substrate to produce a p^+^-i-n^+^ structure. The In_0.5_Ga_0.5_P p^+^ and n^+^ layers had doping concentrations of 2 × 10^18^ cm^−3^. The structure was completed with a highly doped (1 × 10^19^ cm^−3^) p-GaAs layer to facilitate Ohmic contacting. Chemical wet etching techniques, in particular 1:1:1 K_2_Cr_2_O_7_:HBr:CH_3_COOH solution followed by 10 s in 1:8:80 H_2_SO_4_:H_2_O_2_:H_2_O solution, were used to fabricate circular mesa photodiodes with 200 μm and 400 diameters. Sidewall passivation techniques on the processed mesa In_0.5_Ga_0.5_P device were not used. Ti/Au (20 nm/200 nm) and InGe/Au (20 nm/200 nm) contacts were deposited on top of the GaAs top layer and onto the rear of the GaAs substrate to form the Ohmic top and rear contacts, respectively. The top Ohmic contacts had annular shapes; they covered 33% and 45% of the top faces of the 400 μm and 200 μm diameter photodiodes, respectively. The device layers, their relative thicknesses, and materials are summarised in Table 1. The diagram of an In_0.5_Ga_0.5_P mesa device is shown in Fig. 8; the geometry of the top contact is also shown in the figure, the bottom contact covered the whole rear surface of the GaAs substrate.

**Table 1:** Layer details of the In_0.5_Ga_0.5_P photodiode.

Layer	Material	Thickness (μm)	Dopant	Dopant Type	Doping density (cm^−3^)
1	Ti	0.02			
2	Au	0.2			
3	GaAs	0.01	Zn	p^+^	1 × 10^19^
4	In_0.5_Ga_0.5_P	0.2	Zn	p^+^	2 × 10^18^
5	In_0.5_Ga_0.5_P	5	undoped		
6	In_0.5_Ga_0.5_P	0.1	Si	n^+^	2 × 10^18^
7	Substrate n^+^ GaAs				
8	InGe	0.02			
9	Au	0.2

### Data availability

Whilst all data from the study and the findings are contained within the paper, further requests for information may be addressed to the authors.

## References

[1] Nelson RJ, Holonyak N (1976). Exciton absorption, photoluminescence and band structure of n-free and n-doped In_1−x_Ga_x_P. J. Phys. Chem. Solids.

[2] Kuo CP, Vong SK, Cohen RM, Stringfellow GB (1985). Effect of mismatch strain on band gap in III‐V semiconductors. J. Appl. Phys..

[3] Ozaki S, Adachi S, Sato M, Ohtsuka K (1996). Ellipsometric and thermoreflectance spectra of (Al_x_Ga_1−x_)_0.5_In_0.5_P alloys. J. Appl. Phys..

[4] Cromer DT, Liberman D (1970). Relativistic calculation of anomalous scattering factors for X rays. J. Chem. Phys.

[5] Jenkins, R., Gould, R. W. & Gedcke, D. *Quantitative X-ray Spectrometry*, Second Ed. (CRC Press, New York, 1995).

[6] Bertuccio G, Caccia S, Puglisi D, Macera D (2010). Advances in silicon carbide X-ray detectors. Nucl. Instrum. Meth. Phys. Res., Sect. A.

[7] Lioliou G, Meng X, Ng JS, Barnett AM (2016). Temperature dependent characterization of gallium arsenide X-ray mesa pin photodiodes. J. Appl. Phys..

[8] Butera S, Gohil T, Lioliou G, Krysa AB, Barnett AM (2016). Temperature study of Al_0. 52_In_0. 48_P detector photon counting X-ray spectrometer. J. Appl. Phys..

[9] Barth JL, Dyer CS, Stassinopoulos EG (2003). Space, atmospheric, and terrestrial radiation environments. IEEE Trans. Nucl. Sci..

[10] Benkhoff J (2010). BepiColombo—Comprehensive exploration of Mercury: Mission overview and science goals. Planet. Space Sci..

[11] Klingelhofer G, Brukner J, D’uston C, Gellert R, Rieder R (2007). The Rosetta alpha particle X-ray spectrometer (APXS). Space Sci. Rev..

[12] Kocak DM, Dalgleish FR, Caimi FM, Schechner YY (2008). A focus on recent developments and trends in underwater imaging. MTS J..

[13] Lioliou, G. & Barnett, A. Elemental analysis of deep seabed minerals using a prototype GaAs photodiode X-ray fluorescence spectrometer. In prep (2017).

[14] Owens A (2001). Hard X-ray spectroscopy using small format GaAs arrays. Nucl. Instrum. Meth. Phys. Res., Sect. A.

[15] Butera S, Lioliou G, Krysa AB, Barnett AM (2016). Characterisation of Al_0.52_In_0.48_P mesa pin photodiodes for X-ray photon counting spectroscopy. J. Appl. Phys..

[16] Auckloo A (2016). Al_0.52_In_0.48_P avalanche photodiodes for soft **X**-ray spectroscopy. J. Inst..

[17] Barnett AM (2011). The spectral resolution of high temperature GaAs photon counting soft X-ray photodiodes. Nucl. Instrum. Meth. Phys. Res., Sect. A.

[18] Owens A, Peacock A (2004). Compound semiconductor radiation detectors. Nucl. Instrum. Meth. Phys. Res. A.

[19] Loupilov A, Sokolov A, Gostilo V (2001). X-ray Peltier cooled detectors for X-ray fluorescence analysis. J. Radiat. Phys. Chem..

[20] Squillante MR, Entine G (1996). Novel concepts in X-ray and γ-ray detection using compound semiconductors. Nucl. Instrum. Meth. Phys. Res. A.

[21] Owens A (2002). The X-ray response of CdZnTe. Nucl. Instr. and Meth. A.

[22] Egarievwe SU, Chen KT, Burger A, James RB, Lisse CM (1996). Detection and Electrical Properties of Cd_1−x_Zn_x_Te Detectors at Elevated Temperatures. J. X-ray Sci. Technol..

[23] Zappettini, A. *et al*. High energy resolution pixel detectors based on boron oxide vertical Bridgman grown CdZnTe crystals. *IEEE Nuclear Science Symposium and Medical Imaging Conference (NSS/MIC)* (2014).

[24] Owens, A. *Compound semiconductor radiation detectors* (CRC Press, Boca Raton 2012).

[25] Owens A (2007). Hard X-ray detection with a gallium phosphide Schottky diode. Nucl. Instr. and Meth. A.

[26] Owens A (2002). The X-ray response of InP. Nucl. Instr. and Meth. A.

[27] Owens A (2002). The X-ray response of InP: Part B, synchrotron radiation measurements. Nucl. Instr. and Meth. A.

[28] Hubbell JH (1982). Photon mass attenuation and energy-absorption coefficients. Int. J. Appl. Radiat. Is..

[29] Keithley Instruments, Inc, *Model* 6487 *Multi-Frequency LCR Meter Manual*, 6487-901-01 Rev B, (Cleveland 2011).

[30] Hewlett Packard, Model HP 4275A Picoammeter/Voltage Source Reference Manual, 04275–90004, (Tokyo 1990).

[31] Shu GW (2012). Dependence of biasing voltage and illumination power on the built-in electric field of InGaP solar cells. JPN J. Appl. Phys..

[32] Sze, S. M. & Ng, K. K. *Physics of semiconductor devices*, Third Ed. (John Wiley & Sons, New Jersey, 2007).

[33] Bertuccio G, Rehak P, Xi D (1993). A novel charge sensitive preamplifier without the feedback resistor. Nucl. Instrum. Meth. Phys. Res. B.

[34] Shotzig U (2000). Half-life and X-ray emission probabilities of ^55^Fe. Appl. Radiat. Isot..

[35] Lioliou G, Barnett AM (2015). Electronic noise in charge sensitive preamplifiers for X-ray spectroscopy and the benefits of a SiC input JFET. Nucl. Instrum. Meth. Phys. Res. A.

[36] Bertuccio G, Pullia A, De Geronimo G (1996). Criteria of choice of the front-end transistor for low-noise preamplification of detector signals at sub-microsecond shaping times for X-and γ-ray spectroscopy. Nucl. Instrum. Meth. Phys. Res. A.

[37] Gatti E, Manfredi PF, Sampietro M, Speziali V (1990). Suboptimal filtering of 1/*f*-noise in detector charge measurements. Nucl. Instrum. Meth. Phys. Res. A.

[38] Suzuki T, Gomyo A, Iijima S (1988). Strong ordering in GaInP alloy semiconductors; Formation mechanism for the ordered phase. J. Cryst. Growth.

[39] Wei S-H, Zunger A (1990). Band‐gap narrowing in ordered and disordered semiconductor alloys. Appl. Phys. Lett..

[40] Minagawa S, Kondow M (1989). Dependence of photoluminescence peak energy of MOVPE-grown AlGaInP on substrate orientation. Electron. Lett..

